# Microtubule dynamic instability: A new model with coupled GTP hydrolysis and multistep catastrophe

**DOI:** 10.1002/bies.201200131

**Published:** 2013-03-27

**Authors:** Hugo Bowne-Anderson, Marija Zanic, Monika Kauer, Jonathon Howard

**Affiliations:** Max Planck Institute of Molecular Cell Biology and GeneticsDresden, Germany

**Keywords:** catastrophe, cytoskeleton, GTP hydrolysis, microtubule

## Abstract

A key question in understanding microtubule dynamics is how GTP hydrolysis leads to catastrophe, the switch from slow growth to rapid shrinkage. We first provide a review of the experimental and modeling literature, and then present a new model of microtubule dynamics. We demonstrate that vectorial, random, and coupled hydrolysis mechanisms are not consistent with the dependence of catastrophe on tubulin concentration and show that, although single-protofilament models can explain many features of dynamics, they do not describe catastrophe as a multistep process. Finally, we present a new combined (coupled plus random hydrolysis) multiple-protofilament model that is a simple, analytically solvable generalization of a single-protofilament model. This model accounts for the observed lifetimes of growing microtubules, the delay to catastrophe following dilution and describes catastrophe as a multistep process.

## Overview of experimental results

### Microtubules exhibit dynamic instability

**Figure 1:**
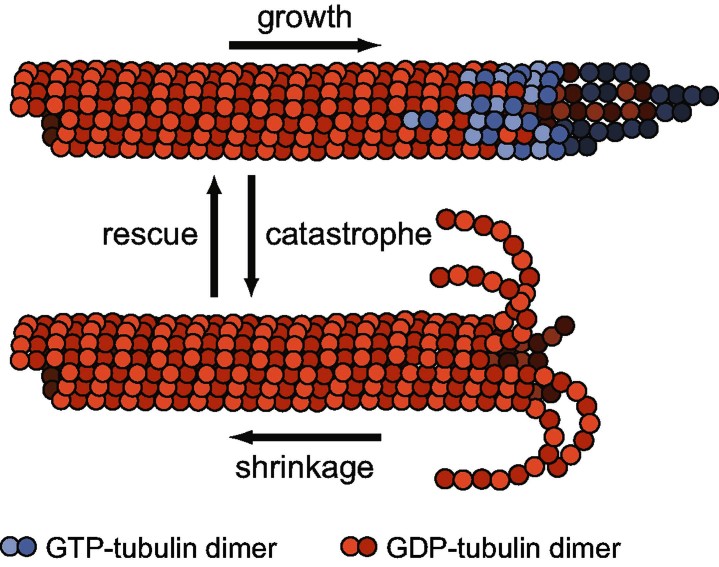
Microtubule dynamic instability. Microtubules are 13-protofilament cylindrical polymers, which switch between phases of growth and shrinkage. Tubulin dimers are incorporated into the growing lattice in the GTP-bound form and stochastically hydrolyze to GDP-tubulin, thus forming a GTP-cap. It is thought that the switching from growth to shrinkage occurs due to the loss of the GTP-cap.

Microtubules are cytoskeletal polymers essential for cell structure, cell division, and intracellular transport. They are typically made of 13 protofilaments, each of which is built from αβ-tubulin dimers. Grown in vitro, microtubules switch between periods of slow growth and rapid shrinkage, behavior known as dynamic instability, discovered by Mitchison and Kirschner 1 (Fig. 1).

Microtubule dynamic instability can empirically be described by four parameters: rate of growth, rate of shrinkage, frequency of switching from growth to shrinkage (known as “catastrophe frequency”) and frequency of switching from shrinkage back to growth (known as “rescue frequency”). As microtubules are polar structures, with their minus ends (exposing α-tubulin) typically anchored in vivo, while their plus ends (exposing β-tubulin) dynamically explore the cellular space, it is of particular interest to study how the four parameters of dynamic instability depend on the tubulin concentration at the microtubule plus end.

Microtubule growth rate is found to scale linearly with tubulin concentration, whereas the shrinkage rate is mostly insensitive to the concentration of tubulin 2. Rescue events are rarely observed in vitro 2 and are not well characterized. Catastrophe events, on the other hand, display low sensitivity with respect to tubulin concentration, such that an increase in tubulin concentration leads to a moderate suppression of microtubule catastrophe at the plus end 2, 3: “the frequency of catastrophe is not steeply dependent on elongation rate” 2. Furthermore, microtubule catastrophe cannot be described as a single-step random process. While growing, microtubules age: younger microtubules have a lower probability of undergoing catastrophe than their older counterparts. This behavior can be understood by viewing microtubule catastrophe as an inherently multistep process 3, 4, leading to a non-exponential distribution of steady-state microtubule lengths unlike the single-step process considered by Verde et al. 5 and Dogterom and Leibler 6.

### Evidence that GTP hydrolysis regulates the catastrophe switch

What powers the dynamic microtubule behavior? The energy required for this process comes from GTP hydrolysis. GTP hydrolysis occurs at the β-tubulin subunit after incorporation of the tubulin dimer into the microtubule lattice. When microtubules are grown with GMPCPP, a non-hydrolyzable form of GTP, they do not undergo dynamic instability 7, showing that GTP hydrolysis is necessary for the switching behavior.

Experiments using laser cutting and micro-needle severing of individual microtubules 8, 52 found that newly exposed plus ends rapidly depolymerize, demonstrating that a growing microtubule has a stabilizing cap on its plus end. Evidence that the stabilizing cap is small comes from dilution experiments 9, 10. In these experiments, microtubules were grown using different tubulin concentrations (leading to different growth rates), and the buffer solution was rapidly exchanged for a solution containing no free tubulin. Microtubules were consequently observed to undergo catastrophe within several seconds upon dilution, much shorter than the time to catastrophe during growth and arguing for a small stabilizing cap whose size does not scale with the growth rate.

It is thought that GTP-tubulin subunits incorporating at the end of a growing microtubule form a GTP-tubulin cap that contributes, somehow, to stability 1. Upon hydrolysis, the GDP-bound microtubule lattice quickly depolymerizes, as the concentration necessary for polymerization of GDP-tubulin is several orders of magnitude higher than that of GTP-tubulin (*K*_c_ in Table 1). However, addition of GMPCPP-tubulin stabilizes microtubules 11 and even a single layer or two of GMPCPP-tubulin is sufficient to prevent depolymerization 12, 13.

**Table 1:** Rate constants for microtubule polymerization and depolymerization at the plus end

Rate constant	GTP-tubulin	GMPCPP-tubulin	GDP-tubulin
*k*_on_ (µM^−1^ s^−1^)	3.2	5.4	–
*k* (s^−1^)	–	0.1	290
*K*_c_ (µM)	0.03^a^	0.02	90^b^

Adapted from Howard 26. Data for GTP/GDP-tubulin comes from Drechsel et al. 36. GMPCPP-tubulin data from Hyman et al. 7. *k*_on_ is the second order association rate constant. *k* is the dissociation rate constant.

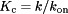
 is the dissociation constant, also called the critical concentration and is the concentration above which there is net growth.

a Assuming the dissociation rate constant for GMPCPP-tubulin.

b Assuming the association rate constant for GTP tubulin.

How big is the GTP-tubulin cap? Although a single layer of GTP-tubulin capping a 13-protofilament microtubule might be sufficient to provide stability (based on the GMPCPP results), the actual size of the GTP cap will ultimately depend on the mechanism of GTP hydrolysis. Tubulin dimers in solution exhibit a low rate of hydrolysis: it is only upon incorporation into the microtubule that GTP hydrolysis is triggered 13. Furthermore, biochemical bulk assays used to determine the rate of GTP hydrolysis in microtubules found little lag between polymerization and hydrolysis 14–16, again arguing for a small GTP cap, as will be discussed more precisely in the modeling section.

The stimulation of GTP hydrolysis by polymerization can arise in several ways, all of which rely on interaction between neighboring dimers in the polymer. Structural studies provide evidence for a specific interaction in which incoming dimers interact with the nucleotides of the terminal dimers at the plus end and trigger their hydrolysis 17. We call this “coupled” hydrolysis to indicate an immediate effect of polymerization on hydrolysis. Alternatively, stimulation could take place when a GTP dimer is more fully incorporated into the lattice and has more neighbors.

Although the GTP cap need only be small, recent high-resolution measurements using optical tweezers observed fluctuations in microtubule growth exhibiting rapid shortening excursions greater than 40 nm (corresponding to five layers of tubulin dimers) without larger-scale microtubule catastrophe 18, 19. This finding implies either a longer GTP cap, or that the lengths of the individual protofilaments can fluctuate (i.e. the end is ragged) and stabilization is conferred at the level where the protofilaments form the tube.

In cells, the dynamic growth and shrinkage of microtubules is regulated by a multitude of microtubule-associated proteins (MAPs). Among them are microtubule polymerases, such as XMAP215, which increase microtubule growth rates up to 10-fold, microtubule depolymerases, such as kinesins from the kinesin-8 and kinesin-13 families, which promote microtubule catastrophe, as well as many plus-end-tracking proteins (+TIPs) known to affect one or more parameters of dynamic instability 20. In this essay, we concentrate on the behavior of tubulin alone because this is a prerequisite for understanding the regulatory effects of MAPs.

How can the existing theoretical models, which assume particular molecular mechanisms of GTP hydrolysis, account for the properties of microtubule dynamic instability? We focus on several experimentally observable parameters. First, we require that a theoretical model reproduces typical lifetimes, that is, the time until catastrophe (several minutes), and lengths (several microns) of microtubules as observed by in vitro experiments for a range of tubulin concentrations 2, 3. Second, we expect that a model replicates the observed moderate suppression of microtubule catastrophe by increasing tubulin concentration 2, 3. Third, we ask that a model predicts the observed non-exponential distributions of microtubule lifetimes 3, 4. Additionally, a successful model should account for microtubule lifetimes observed in dilution experiments 9, 10, as well as for the potential existence of recently observed GTP-tubulin remnants embedded in the microtubule lattice 21.

## Review of existing models

**Figure 2:**
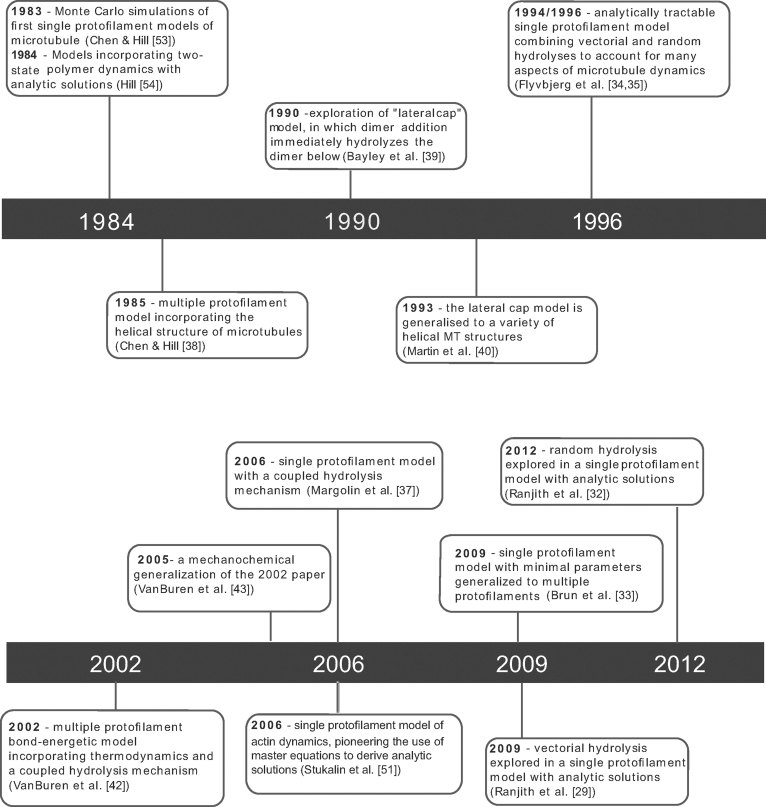
Timeline of milestones in modeling microtubule dynamics.

In the following, we distinguish between three types of models. A conceptual model is a proposed mechanism underlying experimental observations. We may describe such a model mathematically using a system of equations. If we are able to derive analytic solutions from these equations, we call this a mathematical model. Alternatively, we can simulate the behavior of the system and this is known as a computational model. Figure 2 provides a brief historical overview of the modeling of microtubule dynamics over the past 30 years, primarily to give a sense of the movement of the field.

**Figure 3:**
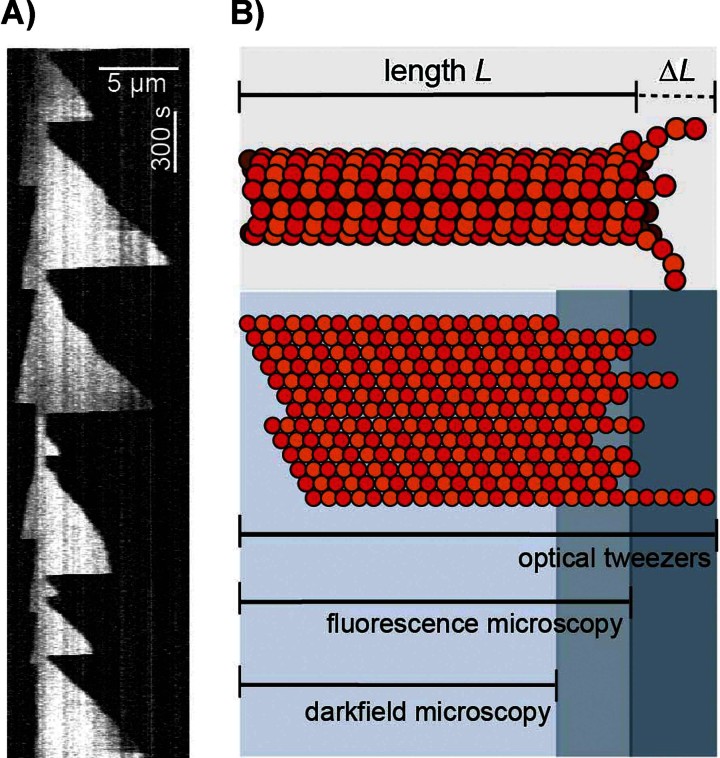
Measuring microtubule lengths and lifetimes. **A:** Kymograph made from a DIC movie, depicting typical microtubule growth and shrinkage using GMPCPP-stabilized microtubule seed and 12 µM tubulin. **B:** The measured length of a microtubule depends on the imaging technique used.

A key parameter associated with microtubule dynamics is length. Given that a microtubule has 13 protofilaments that are not necessarily of the same length, there are several possible definitions of the microtubule end, which will in turn influence the definition of microtubule length. For example, Fig. 3A represents a kymograph depicting typical in vitro growth and shrinkage of a microtubule, imaged by differential-interference-contrast (DIC) microscopy. Because DIC, fluorescence and phase contrast microscopy assay tubulin protein, the length of the microtubule measured by these techniques corresponds to the average protofilament length (Fig. 3B). By contrast, because even one protofilament is expected to be relatively stiff 11, optical tweezers will measure the maximum protofilament length. On the other hand, dark field microscopy is a nonlinear optical technique biasing towards higher mass per unit length and thus will measure more nearly the length of the actual tube.

How ragged is the microtubule end likely to be? Suppose that the growth of each protofilament is an independent Poisson process. Assuming a Poisson distribution of protofilament lengths, for a microtubule with average protofilament length of 8 µm or 1,000 tubulin dimers, the standard deviation is 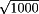 dimers, approximately 280 nm. If longer protofilaments tend to shorten more quickly and the shorter less, the standard deviation is expected to be reduced. Thus we expect all three definitions of microtubule length (Fig. 3B) to be equal on lengths comparable to the wavelength of light, but not necessarily on the nanometer scale. Indeed, observed large and rapid fluctuations in microtubule growth 18, 19, 22 are consistent with a ragged end structure to which subunits bind and unbind on the millisecond timescale. Furthermore, a wide variety of microtubule end structures have been observed by electron microscopy, ranging from blunt, to tapered, to ragged 23, 24. Because the time to catastrophe is on the order of tens to hundreds of seconds, we do not consider the rapid fluctuations in tip length 22, but instead restrict ourselves to timescales corresponding to the stable incorporation of tubulin dimers into the closed lattice.

### What can we learn from single-protofilament models?

The models we consider are kinetic descriptions of microtubule assembly and disassembly. Protein-structure details are discarded and only the essential rate constants are included: the GTP-dimer association rate constant *r* (on-rate), the GTP-dimer dissociation rate constant *k* (GTP-tubulin off-rate), and the hydrolysis rate constant *h*. *h* and *k* are constant while 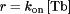 is proportional to tubulin concentration, where *k*_on_ is the second-order association rate constant. The GDP-tubulin off-rate is very high (Table 1) and is thus usually considered to be infinitely large for modeling purposes, though it can be important, as we shall see below. In formulating a single-protofilament model, it is key to map the experimental rates measured for multi-protofilament microtubules onto a single-protofilament model. There are two ways to do so:

1. To view the microtubule as a single protofilament growing helically. In this case, the rate constants of the single-protofilament model remain as measured, while the dimer length in the model is the experimental dimer length divided by 13 (0.6 nm).
2. To consider the microtubule as a collection of 13 individual protofilaments growing simultaneously. Here, each single protofilament will grow with association and dissociation rate constants *r*/13 and *k*/13, respectively.

The former way is most prevalent in the literature. The latter, which takes into account lateral interactions between protofilaments, is how we shall frame the model we propose below.

Although single-protofilament models can explain many experimental observations and are computationally attractive, they are problematic for not taking into account lateral bond interactions. Thus, in single-protofilament models, the likelihood of the microtubule breaking anywhere along its length is the same as that of the terminal dimer dissociating, contradicting the fundamental property of biological polymers that dynamics occurs at polymer ends 25, 26. Considering both this and the fact that single-protofilament models do not explain the multistep nature of catastrophe observed experimentally, we argue that a multiple-protofilament model must be used to understand dynamic instability.

Despite their limitations, there is a great deal to learn from single-protofilament models, not least because they form the basis for our multiple-protofilament model. Therefore, we now provide a review of existing single-protofilament models. There are two key questions in the construction of any model: how the hydrolysis mechanism operates, and how catastrophe is defined. For each model, we first introduce the hydrolysis mechanism and then explore plausible definitions of catastrophe.

#### Coupled hydrolysis

We first consider a coupled model in which a GTP-tubulin dimer hydrolyzes immediately when another is incorporated into the lattice on top of it. This means that the GTP cap is always precisely one dimer deep, and catastrophe is defined to occur when the terminal dimer is GDP-associated. Because the terminal dimer cannot hydrolyze, catastrophe will only occur when it dissociates. Thus, the rate of catastrophe is *k*, the GTP-tubulin off-rate, and the mean lifetime 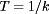 is independent of tubulin concentration. Because this contradicts the experimental data, which shows tubulin concentration dependence 2, 3, 23, a coupled hydrolysis model alone cannot account for dynamic instability.

#### Vectorial hydrolysis

**Figure 4:**
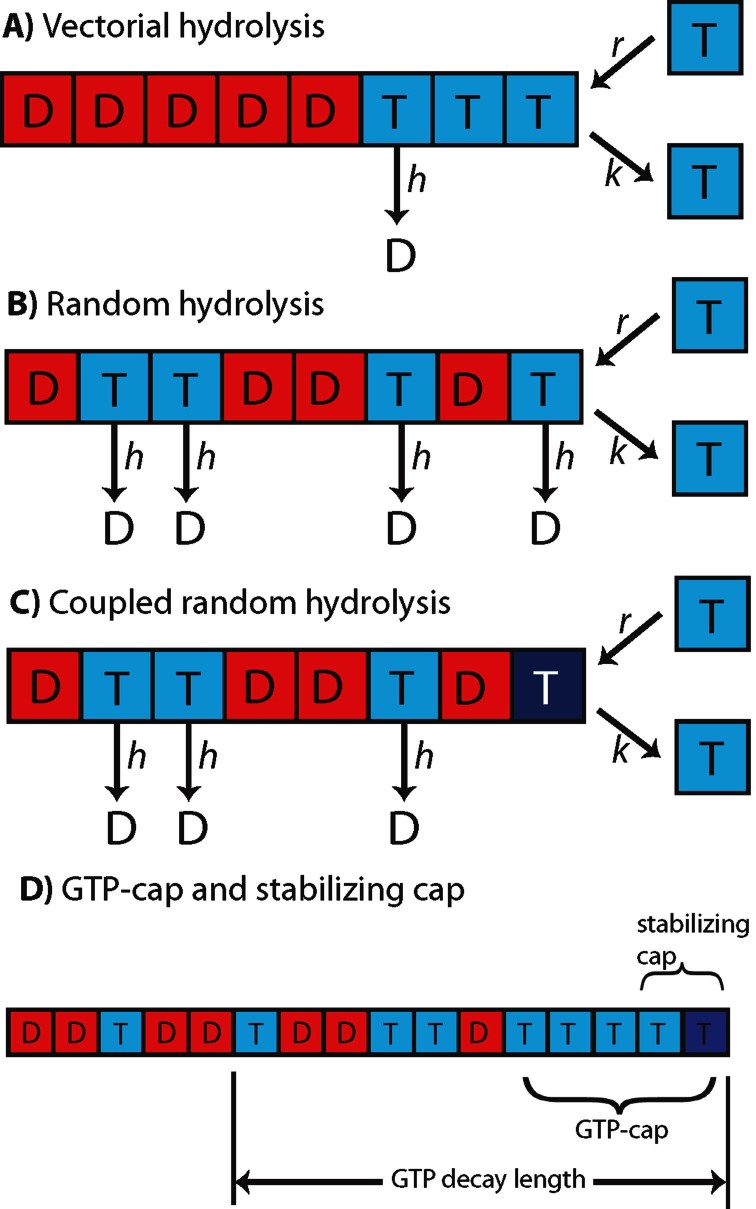
Schematic of single protofilament models. **A:** Vectorial hydrolysis, in which hydrolysis occurs only at the GDP-/GTP-tubulin-interface. **B:** Random hydrolysis, in which at any time, each GTP-tubulin dimer in the microtubule has the same probability of hydrolyzing. **C:** Coupled-random hydrolysis, in which the hydrolysis occurs randomly except that the terminal dimer cannot hydrolyze. **D:** The distinction between the stabilizing cap, the GTP-cap (the length of uninterrupted GTP-tubulin at the end), and the GTP-tubulin decay length, over which the fraction of GTP-tubulin drops *e*-fold.

The mechanism of vectorial hydrolysis may be stated simply: hydrolysis occurs only at the GDP-tubulin/GTP-tubulin interface (Fig. 4A), and catastrophe is defined as the disappearance of the GTP cap. Models incorporating vectorial hydrolysis have been explored most recently in 27–30. The strengths of such mathematical models are that they are simple enough to allow analytic solutions for parameters such as average length, growth velocity, and lifetime, and that they are equivalent to first-passage time problems 29, 31. The main problem with vectorial hydrolysis models is that dynamics are predicted over only a very small range of tubulin concentrations. This follows because either (i) the growth rate will be smaller than the hydrolysis rate and microtubules will catastrophe almost immediately upon nucleation or (ii) the growth rate will be larger than the hydrolysis rate, the cap will be large (and keep getting larger) and there will be hardly any catastrophes at all. Although this was realized by Walker et al. 2 among others, the recent work of Ranjith et al. 29 (using master equation techniques developed in 51) allows a rigorous formulation of this, which we now provide.

**Figure 5:**
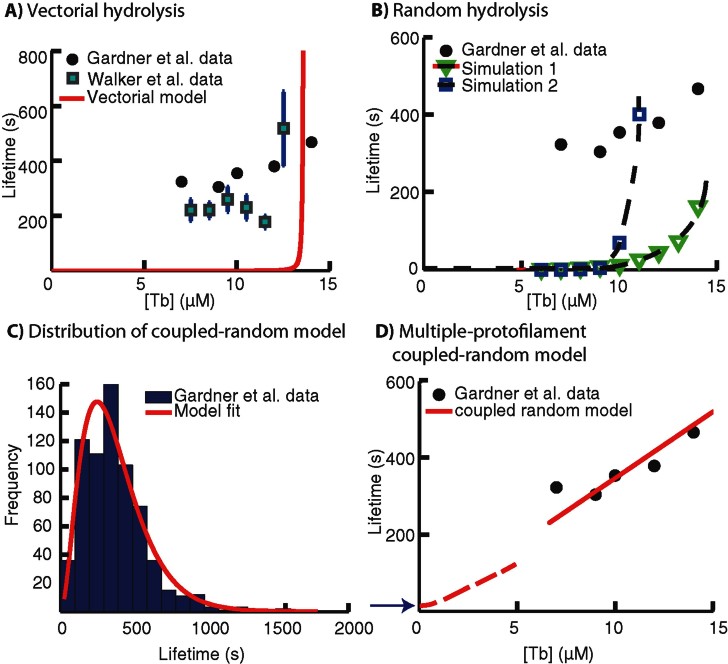
Predictions of the main models alongside experimental data from Walker et al. 2 and Gardner et al. 3. **A:** Vectorial hydrolysis with rates *k*_on_ = 3.2 µM^−1^ s^−1^, *k* = 0.1 s^−1^, and *h* = 43.5 s^−1^. This value of *h* satisfies *h* > *r* − *k* ≍ 42 s. The data from Walker et al. 2 are the points for which the authors had >10 observations. Error bars represent SE. **B:** Random hydrolysis: Simulation 1 rates are *k*_on_ = 3.2 µM^−1^ s^−1^, *k* = 1 s^−1^, and *h* = 1.43 s^−1^; Simulation 2 rates are *k*_on_ = 3.2 µM^−1^ s^−1^, *k* = 24 s^−1^, and *h* = 0.26 s^−1^. **C:** The multiple-protofilament coupled-random model fitted to 12 µM data from Gardner et al. 3. To do so, we used the *mle* MATLAB function. We did so for *n* = 2, 3, 4, with free parameter *T*′. The closest fit was given by *n* = 3 and a step time *T*′ = 1,580 ± 30 s. **D:** Analytic solution to the multiple-protofilament coupled-random model (Equation 2a), fitted to the lifetime data from Gardner et al. 3 and the dilution data from Walker et al. 9 (solid line); *k*_on_ = 3.2 µM^−1^ s^−1^, *k* = 0.2 s^−1^, *h* = 0.12 s^−1^. Microtubules are not observed to nucleate below 5 µM. Our model predicts lifetimes of microtubules when diluted to concentrations above 0 µM and below the critical concentration for growth (dashed line). The arrow points to the predictions for dilution experiments to 0 µM in the model.

We now make the vectorial model precise mathematically: for the average microtubule length to be bounded in the vectorial model, the hydrolysis front, moving at rate *h*, needs to be able to catch up to the microtubule tip, which moves at rate *r*–*k*, implying that 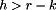. Otherwise, on average, the cap length will either be constant or increase with time and in both these cases the microtubule will not catastrophe. Then, the upper bound on *r* for dynamics to occur is 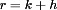. However, it is observed that dynamic instability occurs over at least a twofold range of tubulin concentrations 2, 3, implying dynamics should also be observed at 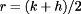. The average microtubule lifetime according to the vectorial model 29 is 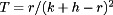, which is 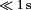 when 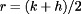, using the value of *k*_on_ from Table 1. Because this is orders of magnitude less than the observed lifetimes (Fig. 5A), a vectorial hydrolysis model alone cannot account for dynamic instability.

#### Random hydrolysis

The basic idea of the random model is that at any time, each GTP-tubulin dimer in the microtubule has the same probability of hydrolyzing, whether embedded in the lattice or at the microtubule end 30, 32 (Fig. 4B). This means that the deeper a dimer is found in the lattice, the greater the probability that it is GDP-associated because it has been in the lattice longer. Two strengths of the random hydrolysis model are that it predicts the existence of GTP islands and small GTP caps. However, the random hydrolysis model suffers from the same problem as the vectorial model: it only predicts dynamics over a small range of tubulin concentrations, although not as small as in the vectorial case.

We have simulated the random hydrolysis model over a range of parameters using a Monte Carlo algorithm. As rescues are rarely observed in vitro, it is natural to define catastrophe as total filament depolymerization. For all parameter sets in our simulations, one of two possibilities occurred, either (i) there was next to no growth at all and very short lifetimes or (ii) microtubule lifetime was far stronger a function of tubulin concentration than what is seen experimentally (Fig. 5B). This is similar to the problem seen in the vectorial model above.

Instead of defining catastrophe as total filament depolymerization, catastrophe could be defined to occur when a number *N* of terminal subunits are GDP-associated 32, 33. For *N* = 1, the lifetime is insensitive to tubulin concentration (see coupled model above). For *N* = 2, the modeled lifetimes exhibit roughly linear increase with tubulin concentration, as experimentally observed.

What is the physical interpretation of *N* = 2? There are two possibilities. The first is that in the time it takes for the terminal two subunits to hydrolyze and dissociate, the GTP-tubulin dimers below them will have hydrolyzed. While this is true if the GDP-tubulin off-rate is slow enough, the high GDP-tubulin off-rates observed are likely to result in rescues when the shrinking microtubule hits a GTP-tubulin dimer in the lattice. Such “mini-catastrophes” 32 would not be visible experimentally as they would be rescued almost straightaway after a shrinkage of only 1.2 nm (two dimers).

Brun et al. 33 suggest another potential explanation for *N*, which they refer to as a “coupling parameter”: any dimers embedded further are hydrolyzed instantaneously. Were *N* = 1, this would be the coupling suggested by the experimental results of Nogales et al. 17 as discussed above. *N* = 2, though, implies that all dimers from the third down are hydrolyzed, meaning that when a dimer is incorporated into the lattice, the one beneath the one it binds to is hydrolyzed, irrespective of the state of the dimer in-between. Such non-nearest-neighbor coupling is difficult to understand mechanistically.

#### Mixed hydrolysis: Vectorial and random

Flyvbjerg et al. 34, 35 mathematically model a single-protofilament with a combination of vectorial and random hydrolyses: there is not only a vectorial boundary moving at the GTP-GDP interface, but every now and then random hydrolysis bisects the GTP cap. This prevents the cap from growing too large. They derive a relatively straightforward analytic approximation for catastrophe frequency, consistent with the data in 36. Their combined model is also in agreement with dilution experiments and predicts the existence of GTP islands, although these islands are relatively short-lived. However, the authors mention explicitly that their model is unable to account for a multistep catastrophe process, as had been observed by Odde et al. 4. This inability to predict multistep catastrophe seems to be the fate of single-protofilament models in general.

#### Mixed hydrolysis: Random and coupled

Margolin et al. 37 present a single-protofilament mathematical model in which hydrolysis occurs randomly, albeit only for those dimers embedded in the lattice and not for the terminal dimer. After calculating the average cap length in their model, the authors consider two ways in which catastrophe may occur: (i) when the average cap length is lost to fluctuations in microtubule growth and (ii) when half the average cap length is hydrolyzed while the other half is lost to fluctuations in growth. They show that in both cases their model is able to predict measured catastrophe frequencies 36. Moreover, the model predicts dilution catastrophe time ≍10 s which is in accordance with experimental data 9. Thus, a coupled-random hydrolysis mechanism can predict key features of microtubule dynamics. Again, the multistep nature of catastrophe is not predicted by this model.

### Multiple-protofilament models incorporate lateral interactions and lead to greater complexity

The first multiple-protofilament model 38 appeared a year after Mitchison and Kirschner's seminal paper 1 and is a computational model. Three years later, Walker et al. 2 wrote “the model of Chen and Hill 38 is perhaps the most complete formal description of cap dynamics … This model can easily fit our catastrophe data for a single end, using only two variations in hydrolysis rate …”

The reason this is true is that the hydrolysis rate of any given subunit depends explicitly on the states of its nearest neighbors, resulting in a large number of potentially different hydrolysis rates (24 in their model). Though they are able to simulate the relatively weak dependence of lifetime as a function of tubulin concentration, the complexity of the model limits its utility for understanding the underlying mechanism.

In 39, 40, we find the first computational model of a coupled mechanism, called the “lateral cap”, in which incorporation of a new subunit immediately hydrolyzes another subunit; in 40, the hydrolyzed subunit is immediately below, while in 39, the hydrolyzed subunit is in a different protofilament. Though coupled hydrolysis in single-protofilament models predicts lifetime to be insensitive to changes in tubulin concentration, the postulated interactions between protofilaments in these multi-protofilament models lead to mean lifetimes that depend so strongly on tubulin concentration that they contradict the experimental data.

VanBuren et al. 41 consider lateral and longitudinal bond energies and determine how these influence microtubule growth and shrinkage rates. Their major insight, partially deriving from 40, is to use bond energetics and experimentally determined dimer association rate constants to infer dimer dissociation rate constants. They utilize a coupled-random computational model, in which hydrolysis occurs randomly in the lattice, but is forbidden for terminal dimers. Although this model reproduced experimental lifetimes at some tubulin concentrations, the authors state that a “shortcoming of the model is that it produces growth lifetimes that more steeply depend on tubulin-GTP concentration than that observed.”

Both VanBuren et al. and Margolin et al. return in 42 and 43, 44, respectively, to generalize their previous models, once again utilizing coupled-random hydrolysis mechanisms. The former account for both mechanical stress and strain, providing a computational model that describes hypothesized microtubule tip structures, while the latter incorporate bond energies and inter-protofilament cracks into their model. In these papers the functional dependence of lifetime on tubulin concentration is not addressed, so it is not known whether these models reproduce the experimental observations. Brun et al. 33 also generalize their single protofilament model to a multiple protofilament setting, utilizing a “gated rescue” mechanism, whereby shrinking protofilaments may be rescued by their neighbors. Their stochastic simulations give rise to exponentially distributed lengths and thus lifetimes, describing catastrophe as a single-step process rather than a multistep one.

## Modeling catastrophe as a multistep process with a coupled-random hydrolysis mechanism

We now present a simple multiple-protofilament model that reproduces the observed microtubule lifetimes and describes catastrophe as a multistep process.

### What is the multistep process?

In our model, we consider a 13-protofilament microtubule as a collection of 13 individual protofilaments each of which has GTP-tubulin association and disassociation rate constants 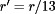 and 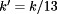, respectively. Hydrolysis in the lattice occurs randomly, but is coupled to tubulin dimer addition: at any time, hydrolysis will occur with rate *h* at any GTP-tubulin dimer, except for the terminal subunit, as in 37, 41 (Fig. 4C). We call this a coupled-random model. Gardner et al. 3 found that microtubule lifetimes can be approximated by a model in which the microtubule catastrophes after a number *n* = 3 of destabilizing events (“steps”). In our model, we interpret a “step” as a permanent modification on an individual protofilament, which occurs when the terminal, stably incorporated subunit is in the GDP state (not considering rapid binding and unbinding of GTP-tubulin, as discussed earlier, p. 6). The step rate in our model occurs with a timescale on the order of microtubule lifetimes, much longer than the rapid tip fluctuations observed in 22. Nevertheless, it is possible that the tip fluctuations could lead to catastrophe, though how these timescales could be bridged is not obvious.

What are the possible mechanistic interpretations for the destabilizing event? One explanation is that once a GDP-tubulin subunit is exposed, incoming GTP-tubulin dimers find it difficult to bind to it, perhaps due to an associated conformational change. This would result in the protofilament no longer growing and thus each destabilizing event would reduce the number of protofilaments by one from that point onward, leading to less stability. Such reductions in protofilament number are seen by electron microscopy 45. Only after three protofilaments have ceased growing will the microtubule catastrophe. Alternatively, the protofilament could continue to grow and those new dimers added immediately hydrolyze, propagating a protofilament consisting entirely of GDP-tubulin subunits, reducing microtubule stability. Either way, the important point is that the destabilization is permanent.

According to the coupled hydrolysis assumption, two criteria must be fulfilled simultaneously for a destabilizing event to occur: (i) the terminal subunit must dissociate from the protofilament and (ii) the subunit below it must be GDP-associated.

In our picture, a protofilament can only destabilize once. We define 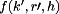 to be the steady state probability that, for a population of growing microtubules 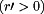, the subterminal subunit of a given microtubule is GDP-associated. Then the average rate of any given protofilament destabilizing is 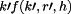, where 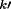 is the dissociation rate constant of the terminal stably incorporated subunit. In this case, the step times are exponentially distributed and the probability distribution function for an individual protofilament's step time is given by 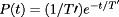, where 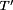, the average step time, is


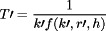
(1)

### Our model predicts observed lifetime distributions

We now calculate the probability of a microtubule undergoing catastrophe, that is, of 3 out of its 13 protofilaments destabilizing. Since the individual protofilaments are considered identical and independent, the probability distribution of waiting times until 3 of 13 destabilize is given by


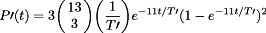
(2)

Using (1) and (2), the average lifetime of a microtubule is:


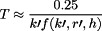
(2a)

Fitting Equation (2) to the experimental data from 3 using a maximum likelihood estimate (MLE), *n* = 3 provided the best fit to the lifetime distribution (Fig. 5C), giving a step time 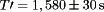, and thus an average lifetime 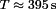.

The computation of 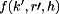 is non-trivial. Fortunately, this is the single-protofilament random-hydrolysis process which has been solved by 32, here applied to the sub-terminal protofilament subunits. We now provide a more easily interpretable approximation. For 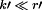, the time evolution of 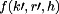 can be described by:


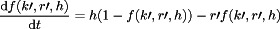


Then, in the steady state, 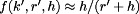. When *h* is small, 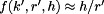. Using (2a):


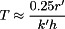
(3)

This accords with an intuitive analysis of the catastrophe process in this model: lifetime scales with dimer association rate constant and scales inversely with both hydrolysis and dissociation rate constants.

### Our model predicts observed dilution lifetimes

To model dilution experiments, we first set the tubulin concentration equal to zero so that 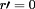. For a step to occur within a protofilament, we require both hydrolysis of the subterminal dimer and dissociation of the terminal one. As GMPCPP is a stable analog of GTP-tubulin, we consider the GMPCPP dissociation rate constant (Table 1) as representative of *k* and then the observed hydrolysis rates are an order of magnitude higher than *k*′ 15, the dissociation rate constant for a single protofilament. In this case 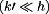, the waiting time for a step is approximated by an exponential distribution with rate constant *k*′ and the zeroth-order approximation of the average step time is 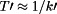. Taking into account that prior to dilution, the population of microtubules has already reached a steady state distribution of steps, the average time to catastrophe after dilution can be calculated to be 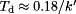. Setting 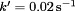 corresponds to a dissociation rate constant 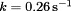, which is of the same order of magnitude as the GMPCPP-tubulin dissociation rate constant (Table 1), and yields a dilution time 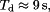 consistent with dilution experiments 9, as well as with reported lifetimes of microtubules grown against barriers 46, assays thought to mimic dilution experiments by preventing GTP-tubulin association.

Taking *T* = 232 s and *r*′ = 3.75 s^−1^ from 2 at 10.5 µM tubulin concentration, *k*′ = 0.02 s^−1^ from the above discussion of the dilution experiments and solving Equation (3) yields *h* = 0.26 s^−1^ consistent with experimental observations (0.1–0.3 s^−1^ 15, ≥0.04 s^−1^ 14). In Fig. 5D, we plot the analytic solution 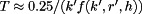 fitted to the lifetime data from 3 and the dilution data from 9. Our model also predicts lifetimes of microtubules when diluted to concentrations above 0 µM given by 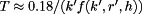 (Fig. 5D, dashed line).

### Our model distinguishes the stabilizing cap from the GTP cap

For a protofilament to destabilize, we require the terminal dimer to dissociate and the one below it to be hydrolyzed; therefore the stabilizing cap for each protofilament is only two dimers deep. This is in agreement with the experimental evidence that the stabilizing cap is small. It is important to realize that our model distinguishes the stabilizing cap from the GTP cap, the latter being the length of uninterrupted GTP-tubulin at the end. The average GTP cap predicted by our model is approximately 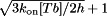 dimers 32, 35, yielding average GTP cap sizes of six and nine dimers on each protofilament, evaluated at 7 µM and 14 µM tubulin, respectively. Thus, the GTP cap might be considerably larger than the stabilizing cap (Fig. 4D). Furthermore, the total number of GTP-tubulin dimers in each protofilament is expected to be even larger. For example, given *h* = 0.2 s^−1^ and a growth rate of 160 nm s^−1^, typically observed in cells, the GTP-tubulin decays to GDP-tubulin over 100 dimers on average. Thus comets of end-binding EB1 proteins found on growing microtubule ends, thought to reflect the nucleotide state of tubulin dimers, may be considerably longer than the stabilizing cap 47–50.

## Conclusion

In 1984, Mitchison and Kirschner 1 discovered microtubule dynamic instability. In the ensuing years, in vitro experiments have explored microtubule dynamics in both pure tubulin and in the presence of MAPs, along with conditions intended to mimic those in cells, such as under force and against barriers. Over these 30-odd years, theorists have been modeling various aspects of microtubule dynamics to varying levels of complexity. These range from coarse-grained mathematical single-protofilament models 34, 35 to computational multiple-protofilament mechanochemical models that account for mechanical stress and strain and bond energetics 41. Despite this, there does not seem to be a consensus among theorists or experimentalists on how the basic kinetic and hydrolysis mechanisms explain what is observed experimentally.

To clarify these issues has been the goal of this paper and to do so has necessarily entailed both a review of the fundamental experimental results in microtubule dynamics and a review of the existing theoretical models. The theoretical models included both single and multiple-protofilament models, and incorporated the most prevalent hydrolysis mechanisms present in the literature: vectorial, random, and coupled. We found that single-protofilament models cannot account for the multistep catastrophe process. Although no existing multiple-protofilament models have been shown to account for all the experimental observations, the models that get closest, such as 41, invoke coupled hydrolysis. Finally, we have presented a simple multiple-protofilament model that utilizes basic kinetics and a coupled-random hydrolysis mechanism. This simple generalization of a single-protofilament model accounts for all the main experimental observations.

## Abbreviations

>DIC: differential-interference-contrast
GDP: guanosine diphosphate
GMPCPP: guanylyl-(α,β)-methylene-diphosphonate
GTP: guanosine triphosphate
MAP: microtubule-associated protein
TIRF: total-internal-reflection-fluorescence
